# Supplementary material for: Relationship between Oral Parafunctional and Postural Habits and the Symptoms of Temporomandibular Disorders: A Survey-Based Cross-Sectional Cohort Study Using Propensity Score Matching Analysis
Source: J Clin Med. 2022 Oct 28;11(21):6396. doi: 10.3390/jcm11216396 (PMC9654264; doi:10.3390/jcm11216396)
Supplement: Supplementary file 1 [file jcm-11-06396-s001.zip › jcm-1968876-supplementary.pdf]

Supplementary Table S1. Demographic and Clinical Characteristics of TMD Patients with and without DC.
（The C–statistic for goodness of fit was 0.709）

Before propensity score matching

		DC		
		Yes (= Presence)	No (= Absence)	*p*–Value
Age (years)		48.0 (31.1 – 62.4) (N = 808)	52.7 (28.1 – 68.2) (N = 1484)	0.003
Gender (N = 2292)	Female	637 (38.5 %)	1016 (61.5 %)	< 0.001
	Male	171 (26.8 %)	468 (73.2 %)	
Medical history of TMJ (N = 2211)	Yes and Same	200 (39.7 %)	304 (60.3 %)	< 0.001
	Yes and Difference	265 (46.7 %)	302 (53.3 %)	
	No	331 (29 %)	809 (71 %)	
TMD treatment (N = 2269)	Yes	240 (45.2 %)	291 (54.8 %)	< 0.001
	No	563 (32.4 %)	1175 (67.6 %)	
Systemic history (N = 2276)	Yes	164 (32.8 %)	336 (67.2 %)	0.167
	No	642 (36.1 %)	1134 (63.9 %)	
Head and neck injury (N = 2277)	Yes	134 (35.8 %)	240 (64.2 %)	0.803
	No	669 (35.2 %)	1234 (64.8 %)	
Orthodontic treatment (N = 2285)	Yes	98 (41.2 %)	140 (58.8 %)	0.047
	No	710 (34.7 %)	1337 (65.3 %)	
Chewing side (N = 2257)	Both side	282 (32.6 %)	583 (67.4 %)	0.082
	Right side	292 (36.2 %)	514 (63.8 %)	
	Left side	223 (38.1 %)	363 (61.9 %)	
Nervous person (N = 2262)	Yes	599 (41.5 %)	843 (58.5 %)	< 0.001
	No	201 (24.5 %)	619 (75.5 %)	
Desk work and recreation (N = 2217)	Yes	488 (39.7 %)	742 (60.3 %)	< 0.001
	No	288 (29.2 %)	699 (70.8 %)	
SB (N = 2292)	Yes	278 (57 %)	210 (43 %)	< 0.001
	No	530 (29.4 %)	1274 (70.6 %)	
CH (N = 2292)	Yes	229 (41.9 %)	317 (58.1 %)	< 0.001
	No	579 (33.2 %)	1167 (66.8 %)	
FD (N = 2292)	Yes	149 (40.3 %)	221 (59.7 %)	0.027
	No	659 (34.3 %)	1263 (65.7 %)	
BP (N = 2292)	Yes	389 (39.1 %)	606 (60.9 %)	0.001
	No	419 (32.3 %)	878 (67.7 %)	

After propensity score matching

		DC		
		Yes (= Presence)	No (= Absence)	*p*–Value
Age (years)		48.3 (30.7 – 63.2) (N = 658)	49.3 (27.4 – 66.5) (N = 658)	0.495
Gender (N = 1316)	Female	506 (49.8 %)	510 (50.2 %)	0.793
	Male	152 (50.7 %)	148 (49.3 %)	
Medical history of TMJ (N = 1316)	Yes and Same	164 (48.2 %)	176 (51.8 %)	0.746
	Yes and Difference	200 (50.4 %)	197 (49.6 %)	
	No	294 (50.8 %)	285 (49.2 %)	
TMD treatment (N = 1316)	Yes	177 (50.4 %)	174 (49.6 %)	0.852
	No	481 (49.8 %)	484 (50.2 %)	
Systemic history (N = 1316)	Yes	138 (48.8 %)	145 (51.2 %)	0.639
	No	520 (50.3 %)	513 (49.7 %)	
Head and neck injury (N = 1316)	Yes	114 (50.2 %)	113 (49.8 %)	0.942
	No	544 (50 %)	545 (50 %)	
Orthodontic treatment (N = 1316)	Yes	80 (50.3 %)	79 (49.7 %)	0.933
	No	578 (50 %)	579 (50 %)	
Chewing side (N = 1316)	Both side	236 (48.8 %)	248 (51.2 %)	0.453
	Right side	239 (49.2 %)	247 (50.8 %)	
	Left side	183 (52.9 %)	163 (47.1 %)	
Nervous person (N = 1316)	Yes	466 (49.1 %)	484 (50.9 %)	0.268
	No	192 (52.5 %)	174 (47.5 %)	
Desk work and recreation (N = 1316)	Yes	402 (51.1 %)	385 (48.9 %)	0.339
	No	256 (48.4 %)	273 (51.6 %)	
SB (N = 1316)	Yes	180 (51.6 %)	169 (48.4 %)	0.492
	No	478 (49.4 %)	489 (50.6 %)	
CH (N = 1316)	Yes	186 (51.5 %)	175 (48.5 %)	0.497
	No	472 (49.4 %)	483 (50.6 %)	
FD (N = 1316)	Yes	122 (52.4 %)	111 (47.6 %)	0.427
	No	536 (49.5 %)	547 (50.5 %)	
BP (N = 1316)	Yes	307 (50.8 %)	297 (49.2 %)	0.580
	No	351 (49.3 %)	361 (50.7 %)	

Supplementary Table S2. Demographic and Clinical Characteristics of TMD Patients with and without DC, if other OPFHs and PHs were absent. （The C–statistic for goodness of fit was 0.682）

Before propensity score matching

		DC		
		Yes (= Presence)	No (= Absence)	*p*–Value
Age (years)		58.9 (44.2 – 67.6) (N = 213)	63.6 (47.1 – 73.2) (N = 586)	0.004
Gender (N = 799)	Female	172 (29.9 %)	404 (70.1 %)	0.001
	Male	41 (18.4 %)	182 (81.6 %)	
Medical history of TMJ (N = 773)	Yes and Same	48 (31.4 %)	105 (68.6 %)	0.001
	Yes and Difference	62 (36 %)	110 (64 %)	
	No	101 (22.5 %)	347 (77.5 %)	
TMD treatment (N = 790)	Yes	62 (33.9 %)	121 (66.1 %)	0.012
	No	149 (24.5 %)	458 (75.5 %)	
Systemic history (N = 796)	Yes	50 (23 %)	167 (77 %)	0.147
	No	163 (28.2 %)	416 (71.8 %)	
Head and neck injury (N = 795)	Yes	32 (28.3 %)	81 (71.7 %)	0.668
	No	180 (26.4 %)	502 (73.6 %)	
Orthodontic treatment (N = 797)	Yes	15 (30.6 %)	34 (69.4 %)	0.526
	No	198 (26.5 %)	550 (73.5 %)	
Chewing side (N = 791)	Both side	70 (21.5 %)	255 (78.5 %)	0.019
	Right side	89 (31.3 %)	195 (68.7 %)	
	Left side	52 (28.6 %)	130 (71.4 %)	
Nervous person (N = 788)	Yes	145 (33 %)	294 (67 %)	< 0.001
	No	66 (18.9 %)	283 (81.1 %)	
Desk work and recreation (N = 774)	Yes	108 (30.8 %)	243 (69.2 %)	0.007
	No	94 (22.2 %)	329 (77.8 %)	

After propensity score matching

		DC		
		Yes (= Presence)	No (= Absence)	*p*–Value
Age (years)		59.1 (44.4 – 67.8) (N = 193)	59.9 (43.9 – 70.2) (N = 193)	0.444
Gender (N = 386)	Female	154 (51.3 %)	146 (48.7 %)	0.328
	Male	39 (45.3 %)	47 (54.7 %)	
Medical history of TMJ (N = 386)	Yes and Same	42 (53.2 %)	37 (46.8 %)	0.819
	Yes and Difference	56 (49.1 %)	58 (50.9 %)	
	No	95 (49.2 %)	98 (50.8 %)	
TMD treatment (N = 386)	Yes	54 (50.9 %)	52 (49.1 %)	0.820
	No	139 (49.6 %)	141 (50.4 %)	
Systemic history (N = 386)	Yes	49 (51.6 %)	46 (48.4 %)	0.723
	No	144 (49.5 %)	147 (50.5 %)	
Head and neck injury (N = 386)	Yes	26 (52 %)	24 (48 %)	0.762
	No	167 (49.7 %)	169 (50.3 %)	
Orthodontic treatment (N = 386)	Yes	11 (47.8 %)	12 (52.2 %)	0.830
	No	182 (50.1 %)	181 (49.9 %)	
Chewing side (N = 386)	Both side	66 (52 %)	61 (48 %)	0.808
	Right side	79 (48.2 %)	85 (51.8 %)	
	Left side	48 (50.5 %)	47 (49.5 %)	
Nervous person (N = 386)	Yes	130 (49.4 %)	133 (50.6 %)	0.743
	No	63 (51.2 %)	60 (48.8 %)	
Desk work and recreation (N = 386)	Yes	99 (50.8 %)	96 (49.2 %)	0.760
	No	94 (49.2 %)	97 (50.8 %)	

Supplementary Table S3. Demographic and Clinical Characteristics of TMD Patients with and without CH.
（The C–statistic for goodness of fit was 0.716）

Before propensity score matching

		CH		
		Yes (= Presence)	No (= Absence)	*p*–Value
Age (years)		33.5 (19.6 – 56.8) (N = 546)	54.8 (34.6 – 68.2) (N = 1746)	< 0.001
Gender (N = 2292)	Female	396 (24 %)	1257 (76 %)	0.808
	Male	150 (23.5 %)	489 (76.5 %)	
Medical history of TMJ (N = 2211)	Yes and Same	135 (26.8 %)	369 (73.2 %)	0.076
	Yes and Difference	145 (25.6 %)	422 (74.4 %)	
	No	252 (22.1 %)	888 (77.9 %)	
TMD treatment (N = 2269)	Yes	130 (24.5 %)	401 (75.5 %)	0.693
	No	411 (23.6 %)	1327 (76.4 %)	
Systemic history (N = 2276)	Yes	86 (17.2 %)	414 (82.8 %)	< 0.001
	No	457 (25.7 %)	1319 (74.3 %)	
Head and neck injury (N = 2277)	Yes	90 (24.1 %)	284 (75.9 %)	0.914
	No	453 (23.8 %)	1450 (76.2 %)	
Orthodontic treatment (N = 2285)	Yes	78 (32.8 %)	160 (67.2 %)	0.001
	No	467 (22.8 %)	1580 (77.2 %)	
Chewing side (N = 2257)	Both side	205 (23.7 %)	660 (76.3 %)	0.454
	Right side	183 (22.7 %)	623 (77.3 %)	
	Left side	150 (25.6 %)	436 (74.4 %)	
Nervous person (N = 2262)	Yes	373 (25.9 %)	1069 (74.1 %)	0.007
	No	171 (20.9 %)	649 (79.1 %)	
Desk work and recreation (N = 2217)	Yes	344 (28 %)	886 (72 %)	< 0.001
	No	189 (19.1 %)	798 (80.9 %)	
SB (N = 2292)	Yes	141 (28.9 %)	347 (71.1 %)	0.003
	No	405 (22.5 %)	1399 (77.5 %)	
CH (N = 2292)	Yes	229 (28.3 %)	579 (71.7 %)	< 0.001
	No	317 (21.4 %)	1167 (78.6 %)	
FD (N = 2292)	Yes	144 (38.9 %)	226 (61.1 %)	< 0.001
	No	402 (20.9 %)	1520 (79.1 %)	
BP (N = 2292)	Yes	338 (34 %)	657 (66 %)	< 0.001
	No	208 (16 %)	1089 (84 %)	

After propensity score matching

		CH		
		Yes (= Presence)	No (= Absence)	*p*–Value
Age (years)		34.5 (19.8 – 57.2) (N = 495)	35.4 (22.9 – 54.5) (N = 495)	0.607
Gender (N = 990)	Female	364 (49.9 %)	366 (50.1 %)	0.885
	Male	131 (50.4 %)	129 (49.6 %)	
Medical history of TMJ (N = 990)	Yes and Same	126 (48.5 %)	134 (51.5 %)	0.835
	Yes and Difference	133 (51 %)	128 (49 %)	
	No	236 (50.3 %)	233 (49.7 %)	
TMD treatment (N = 990)	Yes	123 (50.4 %)	121 (49.6 %)	0.883
	No	372 (49.9 %)	374 (50.1 %)	
Systemic history (N = 990)	Yes	81 (49.4 %)	83 (50.6 %)	0.864
	No	414 (50.1 %)	412 (49.9 %)	
Head and neck injury (N = 990)	Yes	84 (48.6 %)	89 (51.4 %)	0.676
	No	411 (50.3 %)	406 (49.7 %)	
Orthodontic treatment (N = 990)	Yes	69 (49.3 %)	71 (50.7 %)	0.855
	No	426 (50.1 %)	424 (49.9 %)	
Chewing side (N = 990)	Both side	192 (48.9 %)	201 (51.1 %)	0.553
	Right side	168 (52.5 %)	152 (47.5 %)	
	Left side	135 (48.7 %)	142 (51.3 %)	
Nervous person (N = 990)	Yes	334 (50.2 %)	331 (49.8 %)	0.839
	No	161 (49.5 %)	164 (50.5 %)	
Desk work and recreation (N = 990)	Yes	314 (49.8 %)	317 (50.2 %)	0.843
	No	181 (50.4 %)	178 (49.6 %)	
SB (N = 990)	Yes	127 (52.3 %)	116 (47.7 %)	0.417
	No	368 (49.3 %)	379 (50.7 %)	
DC (N = 990)	Yes	206 (50.2 %)	204 (49.8 %)	0.897
	No	289 (49.8 %)	291 (50.2 %)	
FD (N = 990)	Yes	123 (49 %)	128 (51 %)	0.715
	No	372 (50.3 %)	367 (49.7 %)	
BP (N = 990)	Yes	302 (50.4 %)	297 (49.6 %)	0.745
	No	193 (49.4 %)	198 (50.6 %)	

Supplementary Table S4. Demographic and Clinical Characteristics of TMD Patients with and without CH, if other OPFHs and PHs were absent. （The C–statistic for goodness of fit was 0.694.）

Before propensity score matching

		CH		
		Yes (= Presence)	No (= Absence)	*p*–Value
Age (years)		48.3 (18.3 – 66.6) (N = 88)	63.6 (47.1 – 73.2) (N = 586)	< 0.001
Gender (N = 674)	Female	63 (13.5 %)	404 (86.5 %)	0.615
	Male	25 (12.1 %)	182 (87.9 %)	
Medical history of TMJ (N = 645)	Yes and Same	13 (11 %)	105 (89 %)	0.446
	Yes and Difference	21 (16 %)	110 (84 %)	
	No	49 (12.4 %)	347 (87.6 %)	
TMD treatment (N = 667)	Yes	12 (9 %)	121 (91 %)	0.112
	No	76 (14.2 %)	458 (85.8 %)	
Systemic history (N = 671)	Yes	15 (8.2 %)	167 (91.8 %)	0.023
	No	73 (14.9 %)	416 (85.1 %)	
Head and neck injury (N = 671)	Yes	7 (8 %)	81 (92 %)	0.124
	No	81 (13.9 %)	502 (86.1 %)	
Orthodontic treatment (N = 671)	Yes	11 (24.4 %)	34 (75.6 %)	0.018
	No	76 (12.1 %)	550 (87.9 %)	
Chewing side (N = 664)	Both side	32 (11.1 %)	255 (88.9 %)	0.146
	Right side	25 (11.4 %)	195 (88.6 %)	
	Left side	27 (17.2 %)	130 (82.8 %)	
Nervous person (N = 664)	Yes	48 (14 %)	294 (86 %)	0.463
	No	39 (12.1 %)	283 (87.9 %)	
Desk work and recreation (N = 658)	Yes	41 (14.4 %)	243 (85.6 %)	0.365
	No	45 (12 %)	329 (88 %)	

After propensity score matching

		CH		
		Yes (= Presence)	No (= Absence)	*p*–Value
Age (years)		50.1 (18.3 – 66.8) (N = 75)	47.8 (22.9 – 68.5) (N = 75)	0.621
Gender (N = 150)	Female	53 (49.1 %)	55 (50.9 %)	0.716
	Male	22 (52.4 %)	20 (47.6 %)	
Medical history of TMJ (N = 150)	Yes and Same	12 (48 %)	13 (52 %)	0.916
	Yes and Difference	17 (53.1 %)	15 (46.9 %)	
	No	46 (49.5 %)	47 (50.5 %)	
TMD treatment (N = 150)	Yes	11 (50 %)	11 (50 %)	1.000
	No	64 (50 %)	64 (50 %)	
Systemic history (N = 150)	Yes	13 (48.1 %)	14 (51.9 %)	0.832
	No	62 (50.4 %)	61 (49.6 %)	
Head and neck injury (N = 150)	Yes	7 (50 %)	7 (50 %)	1.000
	No	68 (50 %)	68 (50 %)	
Orthodontic treatment (N = 150)	Yes	9 (50 %)	9 (50 %)	1.000
	No	66 (50 %)	66 (50 %)	
Chewing side (N = 150)	Both side	30 (50 %)	30 (50 %)	0.915
	Right side	25 (52.1 %)	23 (47.9 %)	
	Left side	20 (47.6 %)	22 (52.4 %)	
Nervous person (N = 150)	Yes	40 (48.8 %)	42 (51.2 %)	0.743
	No	35 (51.5 %)	33 (48.5 %)	
Desk work and recreation (N = 150)	Yes	34 (51.5 %)	32 (48.5 %)	0.742
	No	41 (48.8 %)	43 (51.2 %)	

Supplementary Table S5. Demographic and Clinical Characteristics of TMD Patients with and without FD.
（The C–statistic for goodness of fit was 0.718）

Before propensity score matching

		FD		
		Yes (= Presence)	No (= Absence)	*p*–Value
Age (years)		32.8 (20.7 – 50.3) (N = 370)	53.8 (32 – 67.7) (N = 1922)	< 0.001
Gender (N = 2292)	Female	251 (15.2 %)	1402 (84.8 %)	0.045
	Male	119 (18.6 %)	520 (81.4 %)	
Medical history of TMJ (N = 2211)	Yes and Same	94 (18.7 %)	410 (81.3 %)	0.247
	Yes and Difference	88 (15.5 %)	479 (84.5 %)	
	No	177 (15.5 %)	963 (84.5 %)	
TMD treatment (N = 2269)	Yes	81 (15.3 %)	450 (84.7 %)	0.551
	No	284 (16.3 %)	1454 (83.7 %)	
Systemic history (N = 2276)	Yes	38 (7.6 %)	462 (92.4 %)	< 0.001
	No	327 (18.4 %)	1449 (81.6 %)	
Head and neck injury (N = 2277)	Yes	68 (18.2 %)	306 (81.8 %)	0.235
	No	299 (15.7 %)	1604 (84.3 %)	
Orthodontic treatment (N = 2285)	Yes	55 (23.1 %)	183 (76.9 %)	0.002
	No	314 (15.3 %)	1733 (84.7 %)	
Chewing side (N = 2257)	Both side	147 (17 %)	718 (83 %)	0.704
	Right side	126 (15.6 %)	680 (84.4 %)	
	Left side	92 (15.7 %)	494 (84.3 %)	
Nervous person (N = 2262)	Yes	249 (17.3 %)	1193 (82.7 %)	0.036
	No	114 (13.9 %)	706 (86.1 %)	
Desk work and recreation (N = 2217)	Yes	247 (20.1 %)	983 (79.9 %)	< 0.001
	No	110 (11.1 %)	877 (88.9 %)	
SB (N = 2292)	Yes	93 (19.1 %)	395 (80.9 %)	0.049
	No	277 (15.4 %)	1527 (84.6 %)	
CH (N = 2292)	Yes	149 (18.4 %)	659 (81.6 %)	0.027
	No	221 (14.9 %)	1263 (85.1 %)	
FD (N = 2292)	Yes	144 (26.4 %)	402 (73.6 %)	< 0.001
	No	226 (12.9 %)	1520 (87.1 %)	
BP (N = 2292)	Yes	213 (21.4 %)	782 (78.6 %)	< 0.001
	No	157 (12.1 %)	1140 (87.9 %)	

After propensity score matching

		FD		
		Yes (= Presence)	No (= Absence)	*p*–Value
Age (years)		31.8 (20 – 49.5) (N = 340)	31.1 (19 – 52.7) (N = 340)	0.839
Gender (N = 680)	Female	229 (50.2 %)	227 (49.8 %)	0.870
	Male	111 (49.6 %)	113 (50.4 %)	
Medical history of TMJ (N = 680)	Yes and Same	90 (49.5 %)	92 (50.5 %)	0.954
	Yes and Difference	82 (49.4 %)	84 (50.6 %)	
	No	168 (50.6 %)	164 (49.4 %)	
TMD treatment (N = 680)	Yes	75 (47.2 %)	84 (52.8 %)	0.415
	No	265 (50.9 %)	256 (49.1 %)	
Systemic history (N = 680)	Yes	35 (48.6 %)	37 (51.4 %)	0.803
	No	305 (50.2 %)	303 (49.8 %)	
Head and neck injury (N = 680)	Yes	63 (50.8 %)	61 (49.2 %)	0.843
	No	277 (49.8 %)	279 (50.2 %)	
Orthodontic treatment (N = 680)	Yes	49 (50.5 %)	48 (49.5 %)	0.913
	No	291 (49.9 %)	292 (50.1 %)	
Chewing side (N = 680)	Both side	139 (50.4 %)	137 (49.6 %)	0.981
	Right side	117 (50 %)	117 (50 %)	
	Left side	84 (49.4 %)	86 (50.6 %)	
Nervous person (N = 680)	Yes	229 (50.1 %)	228 (49.9 %)	0.935
	No	111 (49.8 %)	112 (50.2 %)	
Desk work and recreation (N = 680)	Yes	237 (50.9 %)	229 (49.1 %)	0.509
	No	103 (48.1 %)	111 (51.9 %)	
SB (N = 680)	Yes	86 (51.2 %)	82 (48.8 %)	0.722
	No	254 (49.6 %)	258 (50.4 %)	
DC (N = 680)	Yes	138 (49.1 %)	143 (50.9 %)	0.697
	No	202 (50.6 %)	197 (49.4 %)	
CH (N = 680)	Yes	137 (48.8 %)	144 (51.2 %)	0.586
	No	203 (50.9 %)	196 (49.1 %)	
BP (N = 680)	Yes	194 (50.3 %)	192 (49.7 %)	0.877
	No	146 (49.7 %)	148 (50.3 %)	

Supplementary Table S6. Demographic and Clinical Characteristics of TMD Patients with and without FD, if other OPFHs and PHs were absent. （The C–statistic for goodness of fit was 0.715）

Before propensity score matching

		FD		
		Yes (= Presence)	No (= Absence)	*p*–Value
Age (years)		43.8 (26 – 69.6)<br>(N = 65)	63.6 (47.1 – 73.2)<br>(N = 586)	< 0.001
Gender<br>(N = 651)	Female	45 (10 %)	404 (90 %)	0.962
	Male	20 (9.9 %)	182 (90.1 %)	
Medical history of TMJ<br>(N = 625)	Yes and Same	15 (12.5 %)	105 (87.5 %)	0.613
	Yes and Difference	12 (9.8 %)	110 (90.2 %)	
	No	36 (9.4 %)	347 (90.6 %)	
TMD treatment<br>(N = 643)	Yes	11 (8.3 %)	121 (91.7 %)	0.486
	No	53 (10.4 %)	458 (89.6 %)	
Systemic history<br>(N = 645)	Yes	10 (5.6 %)	167 (94.4 %)	0.036
	No	52 (11.1 %)	416 (88.9 %)	
Head and neck injury<br>(N = 646)	Yes	10 (11 %)	81 (89 %)	0.668
	No	53 (9.5 %)	502 (90.5 %)	
Orthodontic treatment<br>(N = 648)	Yes	8 (19 %)	34 (81 %)	0.039
	No	56 (9.2 %)	550 (90.8 %)	
Chewing side<br>(N = 642)	Both side	27 (9.6 %)	255 (90.4 %)	0.616
	Right side	18 (8.5 %)	195 (91.5 %)	
	Left side	17 (11.6 %)	130 (88.4 %)	
Nervous person<br>(N = 639)	Yes	40 (12 %)	294 (88 %)	0.042
	No	22 (7.2 %)	283 (92.8 %)	
Desk work and recreation<br>(N = 634)	Yes	36 (12.9 %)	243 (87.1 %)	0.019
	No	26 (7.3 %)	329 (92.7 %)	

After propensity score matching

		FD		
		Yes (= Presence)	No (= Absence)	*p*–Value
Age (years)		43.8 (28.1 – 67.5)<br>(N = 55)	54.5 (21.6 – 73.2)<br>(N =55)	0.356
Gender<br>(N = 110)	Female	36 (50 %)	36 (50 %)	1.000
	Male	19 (50 %)	19 (50 %)	
Medical history of TMJ<br>(N = 110)	Yes and Same	11 (42.3 %)	15 (57.7 %)	0.650
	Yes and Difference	12 (54.5 %)	10 (45.5 %)	
	No	32 (51.6 %)	30 (48.4 %)	
TMD treatment<br>(N = 110)	Yes	10 (45.5 %)	12 (54.5 %)	0.634
	No	45 (51.1 %)	43 (48.9 %)	
Systemic history<br>(N = 110)	Yes	10 (40 %)	15 (60 %)	0.255
	No	45 (52.9 %)	40 (47.1 %)	
Head and neck injury<br>(N = 110)	Yes	9 (40.9 %)	13 (59.1 %)	0.340
	No	46 (52.3 %)	42 (47.7 %)	
Orthodontic treatment<br>(N = 110)	Yes	5 (50 %)	5 (50 %)	1.000
	No	50 (50 %)	50 (50 %)	
Chewing side<br>(N = 110)	Both side	26 (53.1 %)	23 (46.9 %)	0.840
	Right side	15 (48.4 %)	16 (51.6 %)	
	Left side	14 (46.7 %)	16 (53.3 %)	
Nervous person<br>(N = 110)	Yes	34 (47.2 %)	38 (52.8 %)	0.423
	No	21 (55.3 %)	17 (44.7 %)	
Desk work and recreation<br>(N = 110)	Yes	31 (48.4 %)	33 (51.6 %)	0.699
	No	24 (52.2 %)	22 (47.8 %)	

Supplementary Table S7. Demographic and Clinical Characteristics of TMJ Patients with and without BP.
（The C–statistic for goodness of fit was 0.713）

Before propensity score matching

		BP		
		Yes (= Presence)	No (= Absence)	*p*–Value
Age (years)		39.3 (23.5 – 58.1) (N = 995)	58.7 (38.9 – 69.8) (N = 1297)	< 0.001
Gender (N = 2292)	Female	719 (43.5 %)	934 (56.5 %)	0.895
	Male	276 (43.2 %)	363 (56.8 %)	
Medical history of TMJ (N = 2211)	Yes and Same	241 (47.8 %)	263 (52.2 %)	0.002
	Yes and Difference	262 (46.2 %)	305 (53.8 %)	
	No	451 (39.6 %)	689 (60.4 %)	
TMD treatment (N = 2269)	Yes	229 (43.1 %)	302 (56.9 %)	0.917
	No	754 (43.4 %)	984 (56.6 %)	
Systemic history (N = 2276)	Yes	183 (36.6 %)	317 (63.4 %)	0.001
	No	804 (45.3 %)	972 (54.7 %)	
Head and neck injury (N = 2277)	Yes	192 (51.3 %)	182 (48.7 %)	0.001
	No	795 (41.8 %)	1108 (58.2 %)	
Orthodontic treatment (N = 2285)	Yes	135 (56.7 %)	103 (43.3 %)	< 0.001
	No	857 (41.9 %)	1190 (58.1 %)	
Chewing side (N = 2257)	Both side	363 (42 %)	502 (58 %)	0.561
	Right side	358 (44.4 %)	448 (55.6 %)	
	Left side	258 (44 %)	328 (56 %)	
Nervous person (N = 2262)	Yes	685 (47.5 %)	757 (52.5 %)	< 0.001
	No	297 (36.2 %)	523 (63.8 %)	
Desk work and recreation (N = 2217)	Yes	594 (48.3 %)	636 (51.7 %)	< 0.001
	No	366 (37.1 %)	621 (62.9 %)	
SB (N = 2292)	Yes	243 (49.8 %)	245 (50.2 %)	0.001
	No	752 (41.7 %)	1052 (58.3 %)	
CH (N = 2292)	Yes	389 (48.1 %)	419 (51.9 %)	0.001
	No	606 (40.8 %)	878 (59.2 %)	
FD (N = 2292)	Yes	338 (61.9 %)	208 (38.1 %)	< 0.001
	No	657 (37.6 %)	1089 (62.4 %)	
BP (N = 2292)	Yes	213 (57.6 %)	157 (42.4 %)	< 0.001
	No	782 (40.7 %)	1140 (59.3 %)	

After propensity score matching

		BP		
		Yes (= Presence)	No (= Absence)	*p*–Value
Age (years)		45.2 (26.7 – 61.2) (N = 689)	46.3 (25.6 – 62.9) (N = 689)	0.785
Gender (N = 1378)	Female	501 (49.8 %)	506 (50.2 %)	0.761
	Male	188 (50.7 %)	183 (49.3 %)	
Medical history of TMJ (N = 1378)	Yes and Same	164 (49.7 %)	166 (50.3 %)	0.991
	Yes and Difference	185 (50 %)	185 (50 %)	
	No	340 (50.1 %)	338 (49.9 %)	
TMD treatment (N = 1378)	Yes	162 (48.9 %)	169 (51.1 %)	0.659
	No	527 (50.3 %)	520 (49.7 %)	
Systemic history (N = 1378)	Yes	144 (49 %)	150 (51 %)	0.693
	No	545 (50.3 %)	539 (49.7 %)	
Head and neck injury (N = 1378)	Yes	125 (50 %)	125 (50 %)	1.000
	No	564 (50 %)	564 (50 %)	
Orthodontic treatment (N = 1378)	Yes	89 (51.7 %)	83 (48.3 %)	0.625
	No	600 (49.8 %)	606 (50.2 %)	
Chewing side (N = 1378)	Both side	268 (50.8 %)	260 (49.2 %)	0.820
	Right side	235 (48.9 %)	246 (51.1 %)	
	Left side	186 (50.4 %)	183 (49.6 %)	
Nervous person (N = 1378)	Yes	460 (51.2 %)	438 (48.8 %)	0.214
	No	229 (47.7 %)	251 (52.3 %)	
Desk work and recreation (N = 1378)	Yes	399 (50.1 %)	397 (49.9 %)	0.913
	No	290 (49.8 %)	292 (50.2 %)	
SB (N = 1378)	Yes	169 (54.5 %)	141 (45.5 %)	0.071
	No	520 (48.7 %)	548 (51.3 %)	
DC (N = 1378)	Yes	276 (52 %)	255 (48 %)	0.245
	No	413 (48.8 %)	434 (51.2 %)	
CH (N = 1378)	Yes	168 (50 %)	168 (50 %)	1.000
	No	521 (50 %)	521 (50 %)	
FD (N = 1378)	Yes	113 (48.7 %)	119 (51.3 %)	0.666
	No	576 (50.3 %)	570 (49.7 %)	

Supplementary Table S8. Demographic and Clinical Characteristics of TMD Patients with and without BP, if other OPFHs and PHs were absent. （The C–statistic for goodness of fit was 0.662）

Before propensity score matching

		BP		
		Yes (= Presence)	No (= Absence)	*p*–Value
Age (years)		51.2 (28.7 – 66.8) (N = 300)	63.6 (47.1 – 73.2) (N = 586)	< 0.001
Gender (N = 886)	Female	202 (33.3 %)	404 (66.7 %)	0.626
	Male	98 (35 %)	182 (65 %)	
Medical history of TMJ (N = 843)	Yes and Same	59 (36 %)	105 (64 %)	0.688
	Yes and Difference	56 (33.7 %)	110 (66.3 %)	
	No	166 (32.4 %)	347 (67.6 %)	
TMD treatment (N = 874)	Yes	50 (29.2 %)	121 (70.8 %)	0.164
	No	245 (34.9 %)	458 (65.1 %)	
Systemic history (N = 879)	Yes	72 (30.1 %)	167 (69.9 %)	0.174
	No	224 (35 %)	416 (65 %)	
Head and neck injury (N = 880)	Yes	54 (40 %)	81 (60 %)	0.095
	No	243 (32.6 %)	502 (67.4 %)	
Orthodontic treatment (N = 881)	Yes	24 (41.4 %)	34 (58.6 %)	0.201
	No	273 (33.2 %)	550 (66.8 %)	
Chewing side (N = 874)	Both side	102 (28.6 %)	255 (71.4 %)	0.028
	Right side	119 (37.9 %)	195 (62.1 %)	
	Left side	73 (36 %)	130 (64 %)	
Nervous person (N = 870)	Yes	178 (37.7 %)	294 (62.3 %)	0.006
	No	115 (28.9 %)	283 (71.1 %)	
Desk work and recreation (N = 857)	Yes	151 (38.3 %)	243 (61.7 %)	0.004
	No	134 (28.9 %)	329 (71.1 %)	

After propensity score matching

		BP		
		Yes (= Presence)	No (= Absence)	*p*–Value
Age (years)		52.4 (29.3 – 68.5) (N = 237)	51 (26.4 – 68.9) (N = 237)	0.669
Gender (N = 474)	Female	164 (50.9 %)	158 (49.1 %)	0.555
	Male	73 (48 %)	79 (52 %)	
Medical history of TMJ (N = 474)	Yes and Same	46 (54.1 %)	39 (45.9 %)	0.656
	Yes and Difference	47 (47.5 %)	52 (52.5 %)	
	No	144 (49.7 %)	146 (50.3 %)	
TMD treatment (N = 474)	Yes	44 (51.8 %)	41 (48.2 %)	0.719
	No	193 (49.6 %)	196 (50.4 %)	
Systemic history (N = 474)	Yes	59 (48.4 %)	63 (51.6 %)	0.674
	No	178 (50.6 %)	174 (49.4 %)	
Head and neck injury (N = 474)	Yes	44 (47.8 %)	48 (52.2 %)	0.642
	No	193 (50.5 %)	189 (49.5 %)	
Orthodontic treatment (N = 474)	Yes	19 (48.7 %)	20 (51.3 %)	0.867
	No	218 (50.1 %)	217 (49.9 %)	
Chewing side (N = 474)	Both side	91 (48.4 %)	97 (51.6 %)	0.853
	Right side	91 (51.1 %)	87 (48.9 %)	
	Left side	55 (50.9 %)	53 (49.1 %)	
Nervous person (N = 474)	Yes	137 (53.1 %)	121 (46.9 %)	0.140
	No	100 (46.3 %)	116 (53.7 %)	
Desk work and recreation (N = 474)	Yes	118 (51.8 %)	110 (48.2 %)	0.462
	No	119 (48.4 %)	127 (51.6 %)	
